# Organotin Compounds Toxicity: Focus on Kidney

**DOI:** 10.3389/fendo.2018.00256

**Published:** 2018-05-22

**Authors:** Carolina Monteiro de Lemos Barbosa, Fernanda Magalhães Ferrão, Jones B. Graceli

**Affiliations:** ^1^Laboratory for Clinical and Experimental Research on Vascular Biology (BioVasc), Department of Physiology, State University of Rio de Janeiro, Rio de Janeiro, Brazil; ^2^Nucleus of Multidisciplinary Research in Biology, Federal University of Rio de Janeiro, Duque de Caxias, Brazil; ^3^Laboratory of Endocrinology and Cellular Toxicology, Department of Morphology, Federal University of Espirito Santo, Vitoria, Brazil

**Keywords:** organotins, kidney, renal function, nephrotoxicity, pollutants, trimethyltin, tributyltin, triphenyltin

## Abstract

Organotin compounds (OTs) are synthetic persistent organometallic xenobiotics widely used in several commercial applications. They exert well-described harmful effects in brain, liver, adipose tissue, and reproductive organs, as they are endocrine-disrupting chemicals (EDCs), but the effects in the kidneys are less known. The kidneys are especially vulnerable to environmental contaminants because they are a metabolizing site of xenobiotics, therefore, pollutants can accumulate in renal tissue, leading to impaired renal function and to several renal abnormalities. Individuals chronically exposed to OTs present a threefold increase in the prevalence of kidney stones. These compounds can directly inhibit H^+^/K^+^-ATPase in renal intercalated cells, resulting in hypokalemia, renal tubular acidity, and increased urinary pH, which is a known risk factor for kidney stones formation. OTs effects are not only limited to induce nephrolithiasis, its nephrotoxicity is also due to increased reactive oxygen species (ROS). This increase leads to lipid peroxidation, abnormal cellular function, and cell death. Combined, the enzymatic and non-enzymatic antioxidant defense systems become deficient and there is a consequent uncontrolled generation of ROS that culminates in renal tissue damage. Still, few epidemiological and experimental studies have reported renal impact correlated to OTs exposure. This lack of investigation of the complete effect of OTs in renal function and structure led us to perform this review reporting the main researches about this subject.

## Introduction

Organotin compounds (OTs) are synthetic organometallic chemicals with several commercial applications. The major one is in the plastics industry which utilizes these compounds particularly to produce polyvinyl chloride (PVC) (1). As PVC polymer is unstable under heat and light, OTs derivatives can be added as stabilizers (2). Methyltin stabilizers are made from monomethyltin (MMT) and dimethyltin (DMT) that are synthesized by a direct chemical reaction. Trimethyltin (TMT) is produced as a byproduct during this synthesis and it is more toxic than MMT and DMT. Methyltin-stabilized PVC is used in packaging, piping, coating, and window frames (1). OTs have been found to leach from PVC pipes and it can contaminate foodstuffs, beverages, drinking water, and sewage (3, 4).

Trisubstituted organotin species have biocidal properties and can be used as agricultural pesticides, wood preservatives, and antifouling paints on ships (5). The broad utilization of OTs allows sizable amounts of them to enter various ecosystems. Specially, tributyltin (TBT) and triphenyltin (TPT) have high complex toxic effect to aquatic life even at low levels (6, 7). They can act as endocrine-disrupting chemicals (EDC) in target and non-target organisms (8). In mollusks, TBT is able to lead to imposex development, which is an abnormal endocrine syndrome with imposition of male sex characteristics in female organisms (9). In mammals, abnormalities in metabolism and in neural, immune, hepatic, and reproductive systems are reported after TBT exposure (10–12). Widespread environmental contamination of marine ecosystems with TBT began in the 1960s and its use in antifouling ship paints was prohibited by the International Marine Organization in 2008 (13, 14). However, beyond its regular use in agriculture and other industrial processes, it is possible that TBT is still used in some parts of the world in countries that are not included in the International Convention on the Control of Harmful Anti-Fouling Systems on Ships and/or with poor environmental monitoring and fiscalization (15, 16).

For the human population, the major route of exposure to most OTs is ingestion, through the consumption of food and/or drinks either contaminated with OTs (17). Marine fishery products may contain high TBT levels (18), and different diets are expected to result in different OTs loads in human tissues (19–21). However, despite the evidence that such sources expose humans to OTs, limited data on deposition in humans are available. Thus, human risk assessment has mainly been based on experimental immunological studies and estimated human intake of seafood sources (18). OTs are detected in human blood at levels that range from 64 to 155 ng/mL, which leads to TBT tissue accumulation and immunological dysfunctions (22).

The impact of methyltin compounds on human health is primarily focused on its neurotoxic effects (23–27). Although the neurotoxic outcomes of OTs have been well documented, their nephrotoxic effects have received little attention. In 1987, Robertson and colleagues described TMT nephrotoxic effects and highlighted how undetected they were until then (28).

The kidneys play important roles in the maintenance of body homeostasis, such as regulation of extracellular fluid osmolality, volume, electrolytes, and acid–base balance (29). Furthermore, kidneys possess most of the common xenobiotic metabolizing enzymes contributing to the metabolism of drugs and foreign compounds, including environmental contaminants (30). In consequence, the kidneys tend to be more susceptible to those substances (31). Indeed, renal xenobiotic exposure leads to improper renal function (32). In this review, the renal outcomes related to OTs will be explored.

## Organotins Induce Structural and Functional Changes in Kidneys

Organotin compounds are acknowledged for its neurotoxic effects, producing a range of neurological symptoms and they are also known for its toxicity in liver and reproductive system (33–35). However, few studies have reported the renal toxicity of these compounds. In 1985, Dwivedi and collaborators were trying to discover the biological effects of OTs and demonstrated that several of these compounds can affect renal enzymatic activities in rats (36). The same study also demonstrated enzymatic alterations in liver and brain (36). Before that, hydronephrosis and vacuolar degeneration of renal tubules were described in rats exposed to TMT (37). Blood urea nitrogen (BUN) levels, tubular dilatation, and epithelial vacuolization were also shown to be increased by TMT exposure (38). However, these reports were conflicting with studies that described no significant effects in the kidneys that could be correlated to OTs exposure (39, 40).

Trimethyltin was first described as a potent nephrotoxicant in two studies where this compound was orally administered in rats (28, 41). TMT induced rats to a renal failure and there was a time-course relationship between the effects on the kidney and various neurological manifestations (28). TMT initially induced oliguria and renal lesions that progressed to acute renal failure. Proteinuria, increase in urinary pH and in BUN levels were also present (28). Histological abnormalities were observed as tubular focal effects especially in the outer medullary area with interstitium expansion and consequent obstruction of the vascular supply and swelling in the renal papilla. Another study demonstrated marked proximal tubular damage with dilation and loss of the brush borders. There was no clear evidence of glomerular damage, thus tubular lesions seem to be more important (28, 41).

Until 1993, neither nephrotoxicity nor pathological changes of the kidney induced by OTs had been reported in human studies. A case study of three patients admitted with acute TPT intoxication showed an increase in serum creatinine and BUN levels, which were compatible with the dysfunctional results of animal studies (42). A significant increase in proteinuria in all three patients could indicate severe tubular and mild glomerular injury (42).

Another important OT that has been investigated is TBT. Low subchronic oral doses of TBT exposure (2.0 or 6.0 µg/kg) were administered to rats once a week for over 30 or 60 days and showed no effect on kidney morphology (43). On the other hand, a higher dose of TBT (50 mg/kg diet) on a 30-month chronic toxicity study in rats resulted in decreased renal function weight (44). Despite the studies about TBT harmfulness, only a few studies evaluated its effects in renal morphology (45, 46). Mitra et al. showed morphological alterations in rats with a low and unique dose of TBT (5 mg/kg): the glomeruli appeared swollen with increased capsular space. Although kidney function was unaltered in this particular study, the oxidative stress, as well as reactive oxygen species (ROS), was increased in renal tissue (34). Thus, TBT presents a complex and contradictory toxicological renal effect. Furthermore, TBT was shown to lead to a reduced glomerular filtration rate (GFR) and increased proteinuria levels in female rats exposed to TBT (100 ng/kg/day) for 15 days (47). Renal structural abnormalities such as increased glomerular tuft area and tubulointerstitial collagen deposition were also observed. Additionally, TBT led to tin renal tissue accumulation associated with higher renal oxidative stress and apoptosis levels, leading to abnormal renal function (47).

Other striking features regarding OTs and their effects in the kidneys are the increase in oxidative stress, hypokalemia state, and kidney stones formation (47–49). They all will be discussed along these lines.

## Organotins Challenge Kidneys with Oxidative Stress

Organotin compounds have many biological impacts and are associated with endocrine and physiologic disruptor effects, acting as EDCs (50). It was recently demonstrated that they are able to bind nuclear receptor, such as glucocorticoid receptors and retinoid X receptor subtypes, forming a complex OTs-nuclear receptors with coactivators and inducing transcription of target genes (51, 52). This process promotes changes in the expression of proteins in addition to mitochondrial and cell dysfunctions (51, 52).

Oxidative stress is the main pathway involved in tissue damage induced by OTs in different organs, such as kidneys, testis, liver, lungs, adrenal gland, pituitary, and brain (34, 47, 53–55). It was described that TBT induces ROS production, lipid peroxidation, and cell death in rodent models (56). Moreover, it decreases the enzymatic and non-enzymatic antioxidant defense systems (catalase, superoxide dismutase, glutathione peroxidase, and vitamins C and E) (53). Indeed, oral administration of TBT for 65 days in Syrian hamsters led to high levels of serum creatinine, urea, bilirubin, and uric acid, with histopathological abnormalities in the testis, liver, and kidneys (53). TBT treatment induced a decrease in the activity of catalase, superoxide dismutase, glutathione peroxidase, and vitamins C and E, and an increase in lipid peroxidation in the same organs (53), demonstrating critical oxidative stress-induced damage by TBT action. ROS generation induced by TBT impairs cell function and culminates in tissue damage (53). Coutinho et al. (47) showed important renal function impairment induced by TBT in female rats, with renal inflammation and fibrosis, increased glomerular tuft area, reduced GFR, and increased proteinuria. TBT effects on renal dysfunction were shown to be due to the oxidative stress and apoptosis levels (47). TBT induced increases in ROS levels in the serum, liver, lung, and kidney of male Wistar rats after subchronic exposure to low doses of TBT for 1 month. In this case, kidney presented a 1.4-fold increase in the ROS levels after 1 mg/kg of TBT for 30 days, showing an important association between TBT exposure and renal ROS development (54).

Likewise the kidney, brain, and cardiovascular system damage induced by OTs are also due to ROS production (34, 55, 57, 58). Neurodegeneration in rats was shown to be *via* oxidative damage, mitochondrial membrane depolarization, DNA damage, and apoptosis in cortical cells, due to ROS overproduction (34). Hippocampus and hypothalamus in rats exposed to TBT develop inflammation, fibrotic process also due to increased oxidative stress (55, 59). Fibrosis also occurs in aortic rings as a consequence of oxidative stress increase induced by TBT exposure in female rats for 15 days (100 ng/kg/day), resulting in functional and morphological dysfunctions (58, 60, 61).

Studies of TBT effects in the kidney suggested that the oxidative stress is the main cause of renal damage induced by OTs (62, 63). Increased ROS induce mitochondrial dysfunction, caspase activation, DNA damage, and cell death, which in turn lead to an irreversible renal dysfunction. Similarly, a common feature of TBT toxicity on brain, testis, liver, lungs, adrenal gland, pituitary, and cardiovascular system was also shown to be due to ROS production (53–55).

Increased oxidative stress induced by OTs can bring damages to kidneys. Those compounds can affect kidneys in other ways like inducing hypokalemia that will be discussed next.

## Organotins are Potent Hypokalemic Inductors

Hypokalemia is a condition of low blood potassium (K^+^) levels and it is one of the most common and dangerous electrolyte abnormalities observed in clinical medicine. It alters the functions of several organs, such as muscles, kidneys, and cardiovascular and neurologic systems (64). Clinical analysis of 76 cases from 13 poisoning accidents caused by TMT demonstrated that 81.6% (62 cases) presented hypokalemia, which persisted for more than 1 week in most cases (23). Urine K^+^ levels were between 5 and 165 mmol/L in 47 patients. Accordingly, other clinical analysis of TMT intoxication also revealed low serum K^+^ levels in 85.7% of the cases (48 from 56 patients) (65). These studies suggested that hypokalemia could be the main clinical indication of TMT intoxication (23, 65). Since no diarrhea or vomiting was observed in TMT intoxicated patients, which could justify blood K^+^ levels reduction, it was postulated that TMT-induced hypokalemia could be due to its leakage in urine (48). Likewise, Guo et al. (63) analyzed 15 patients that were admitted to Sir Run Run Shaw Hospital from 2002 to 2007 with OTs poisoning and observed that most of patients presented elevated blood ammonia, metabolic acidosis, and decreased K^+^ blood levels, as a result of renal OTs toxicity.

**Figure 1:**
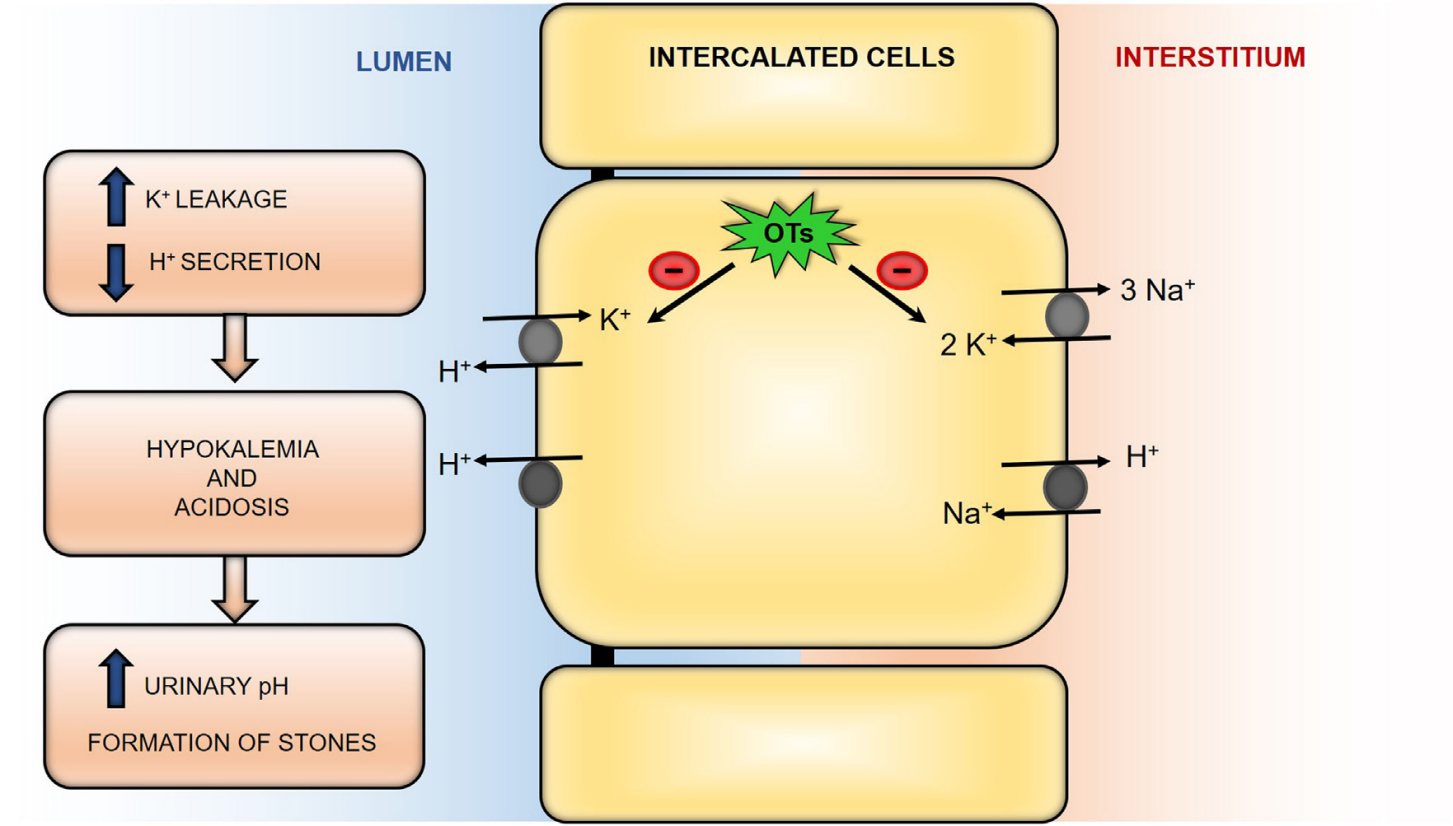
Schematic representation of organotin compounds (OTs) effects in renal intercalated cells in mammalian models. OTs are able to inhibit both H^+^/K^+^-ATPase and Na^+^/K^+^-ATPase activities. This inhibition leads to hypokalemia and acidosis. Former is due to increased K^+^ leakage to the lumen, and latter is due to decreased H^+^ secretion. In consequence, urinary pH is increased, which is a known factor for formation of kidney stones.

Unbound K^+^ is freely filtered across the glomerulus and the majority of tubular K^+^ is reabsorbed along the proximal tubule and the thick ascending limb of Henle’s loop (66). Only 10% of filtered K^+^ reaches the distal nephron and generally 10–20% of the filtered K^+^ load is excreted (67), suggesting that this ion is secreted along the nephron. The control of K^+^ secretion within the kidney occurs in the distal nephron (68). The collecting duct is composed of two cell types: principal and intercalated cells. Intercalated cells represent a small fraction of epithelial cells along the distal nephron that reabsorb K^+^ *via* luminal-membrane H^+^/K^+^-ATPase. Inhibition of this ATPase prevents K^+^ reabsorption and H^+^ secretion and was suggested to be the mechanism underlying TMT-induced hypokalemia (48). Indeed, administration of 10 mg/kg of TMT in Sprague-Dawley rats induced hypokalemia and as K^+^ serum levels were decreased, the K^+^ leakage in the urine was increased (48). Although H^+^/K^+^-ATPase mRNA content and expression was not changed, TMT inhibited intercalated cells H^+^/K^+^-ATPase activity and K^+^ reabsorption, decreasing H^+^ secretion, inducing hypokalemia and acidosis, as reported in Figure 1 (48).

It was demonstrated that other ATPase is involved with TMT-induced hypokalemia (48). Sprague-Dawley rats treated with 10 or 21.5 mg/kg of TMT for 11 days had a rapid and persistent decrease in plasma K^+^ level, starting 30 min after the treatment and persisting until the end of the experiment at the 11th day. It was suggested that Na^+^/K^+^-ATPase modulation is the cause of TMT-induced hypokalemia, since its activity was decreased after TMT treatment, reducing renal K^+^ reabsorption (48). Accordingly, Sprague-Dawley rats treated with 10 mg/kg of TMT presented a decrease in plasma K^+^ level with the lowest dosage on day 6 (4.85 mmol/L) and recovering on day 28, and this TMT-induced hypokalemia was accompanied by Na^+^/K^+^-ATPase activity decrease (69). It was suggested that a rise of plasma aldosterone levels plays an important role on K^+^ leakage resulting in TMT-induced hypokalemia, since it was tenfold increased after exposing rats to 46.4 mg/kg of TMT (69).

Not only TMT, but also other OTs are able to induce hypokalemia. Sprague-Dawley rats and Chinese Kun Ming mice treated *via* gavage and intraperitoneal injection with both DMT and TMT, presented a significant decrease in plasma K^+^ level after 1 h of treatment, and the effect persisted for over 7 days (70). Both animal studies and clinical analyses of poisoned patients, demonstrated that OTs (TMT mostly) are powerful hypokalemia inductors, leading to decreased plasma K^+^ levels, H^+^ secretion, and consequent renal tubular cells acidity and urinary pH increase. This alkaline environment as result of OTs exposure favors the formation of various types of kidney stones and will be discussed in the next topic.

## Exposure to Organotins is Associated with Kidney Stones

Kidney stones are a common health problem in industrialized countries affecting around 2–5% of the population during lifetime at least once (62, 71). The prevalence and incidence of nephrolithiasis is reportedly being increased globally. In the United States, overall stones prevalence has doubled over the past three decades. This increase has also been noted in most European countries and Southeast Asia (72, 73). The cause of these changes is unclear, but many factors predispose or contribute to the development of kidney stones, including genetic factors, diet, behavior, and environmental factors. It has been suspected that the latter is a potential major contributing cause. OTs such as TMT provoke hypokalemia likely due to H^+^/K^+^-ATPase inhibition which leads to urinary pH increase, as exposed above (48). The disruption of urinary pH and alteration of electrolytes levels may promote crystal deposition and stones formation in kidneys and urinary tract, as displayed in Figure 1 (74).

As TMT potentially can induce nephrolithiasis, Tang et al. (70) examined 216 manufacturing workers exposed to TMT for at least 3 months and 119 control individuals that worked at the plant, but unexposed. Workers exposed to relative low levels of TMT in the air (<0.013 mg/m^3^) were more likely to have kidney stones (threefold higher than the control group) and it was prevalent especially among those who were employed longer. This suggests a renal toxic effect that chronical TMT exposure may cause (70).

Another study investigated the long-term effect of TMT (14.7 mg/kg) submitting rats to this organotin in the drinking water for 6 months (49). It was shown that different levels of TMT could induce a dose-dependent increase in kidney stones formation. As described in human poisoning cases, rats also presented inhibition of renal H^+^/K^+^-ATPase activity which leads to urinary pH increase. Alkaline urine favors the formation of calcium and phosphate stones; struvite stones can occur when urine pH is neutral or alkaline. TMT-treated rats presented the majority of stones composed of struvite in addition to calcium components, while the control group did not present any stones (62, 71).

Taking all these evidences in consideration, TMT exposure is positively associated with the development of kidney stones. The rising presence of this OTs in our daily environment may contribute to increase the risk of developing kidney stones and/or other renal abnormalities. Additional and comprehensive studies are necessary to corroborate with these findings and shed light on this subject.

## Conclusion

Organotin compounds are a threat to human health and they are broadly used with various agricultural and industrial applications. Although their harmful effects have been better described in liver, reproductive, and nervous systems, their effects in the kidneys have not been widely investigated. The published data indicate that those contaminants have an impact in the kidney proper functioning, mostly, on its oxidative stress damage, on hypokalemia induction and on kidney stones formation. OTs toxicity depends on concentration, time of exposure, as well as the kind of species that it is being exposed. Therefore, OTs lead to an important renal toxicity that can be considered an important environmental risk for renal diseases development.

## Author Contributions

CB and FF contributed for data review and text writing. Editing and critical analysis were done by all three writers (CB, FF, and JG). CB prepared the figure.

## Conflict of Interest Statement

The authors declare that the research was conducted in the absence of any commercial or financial relationships that could be construed as a potential conflict of interest.
